# Antagonistic and additive effect when combining biopesticides against the fall armyworm, *Spodoptera frugiperda*

**DOI:** 10.1038/s41598-024-56599-w

**Published:** 2024-03-12

**Authors:** Steven J. Harte, Daniel P. Bray, Victoria Nash-Woolley, Philip C. Stevenson, G. Mandela Fernández-Grandon

**Affiliations:** 1 Natural Resources Institute, University of Greenwich, Central Avenue, Chatham Maritime, Kent, ME4 4TB UK; 2 CHAP, Innovation Centre, Innovation Way, Heslington, YO10 5DG UK; 3 Royal Botanic Gardens, Kew, Kew Green, Richmond, Surrey TW9 3AE UK

**Keywords:** Biopesticide, Botanicals, Pyrethrum, Entomopathogenic Fungi, Invasive species, Fungi, Chemical biology, Natural products

## Abstract

Fall armyworm, *Spodoptera frugiperda* (FAW) is a cosmopolitan crop pest species that has recently become established in sub-Saharan Africa and Southeast Asia. Current FAW control is almost entirely dependent on synthetic pesticides. Biopesticides offer a more sustainable alternative but have limitations. For example, pyrethrum is an effective botanical insecticide with low mammalian toxicity but is highly UV labile, resulting in a rapid loss of efficacy in the field. *Beauveria bassiana* is an entomopathogenic fungus that is more persistent, but there is a time lag of several days before it causes insect mortality and leads to effective control. The combination of these biopesticides could mitigate their drawbacks for FAW control. Here we evaluated the efficacy of pyrethrum and *B. bassiana* as individual treatments and in combination against 3^rd^ instar FAW. Four different combinations of these two biopesticides were tested, resulting in an antagonistic relationship at the lowest concentrations of *B. bassiana* and pyrethrum (1 × 10^4^ conidia mL^−1^ with 25 ppm) and an additive effect for the other 3 combined treatments (1 × 10^4^ conidia mL^−1^ with 100 ppm and 1 × 10^5^ conidia mL^−1^ with 25 ppm and 100 ppm pyrethrum). Additionally, a delay in efficacy from *B. bassiana* was observed when combined with pyrethrum as well as a general inhibition of growth on agar plates. These results appear to show that this particular combination of biopesticides is not universally beneficial or detrimental to pest control strategies and is dependent on the doses of each biopesticide applied. However, the additive effect shown here at specific concentrations does indicate that combining biopesticides could help overcome the challenges of persistence seen in botanical pesticides and the slow establishment of EPF, with the potential to improve effectiveness of biopesticides for IPM.

## Introduction

Fall armyworm, *Spodoptera frugiperda* (J.E. Smith) (Lepidoptera: Noctuidae) (FAW) is a cosmopolitan crop pest species that has been a long-established pest in the Americas^1^ but has only recently become a global challenge for food production. Native to Central America, the species has spread to Canada and Argentina^2^. In Africa, it was first recorded in Nigeria in January 2016^3^. It is now found in over 44 other African countries and countries in South and East Asia including India, Nepal, Bangladesh and China^4–7^.

Crop yield losses from FAW vary, but Kumelaa et al.^8^ reported maize losses of up to 40–70% in Kenya and 40–55% yield in Ethiopia, with 97% of farmers in Kenya reporting some losses from FAW and 93% in Ethiopia. Other reports from sub-Saharan Africa support these findings with similar losses due to FAW crop damage found^9–11^. Early detection of FAW is crucial for effective control and damage mitigation because early instars are more susceptible to most control measures^12^. However, FAW is difficult to distinguish from other Spodoptera species such as beet armyworm (*Spodoptera exigua*) and African armyworm (*Spodoptera exempta*), which are native to Africa and Asia^13^. The rapid spread of FAW is aided by their ability to fly over 60 km per night^14^. This spread of FAW is such that it is now considered a global threat, with the EU issuing commission Decision (EU) 2018/638 (2018) for emergency measures to prevent the introduction into, and the spread of FAW within, the EU^15^.

Typically, FAW control has been achieved using synthetic pesticides. However, this can require higher volume applications than those needed for other pest species^12,16^, often requiring multiple applications to manage established FAW populations^17^. This excessive use of synthetic pesticides has led to resistance against pyrethroids, organophosphates and carbamates^18–20^. This control model is responsible for substantial environmental impacts, diminishing beneficial insect populations, and threatening food safety.

Environmentally benign pest management options for FAW control exist, however FAW has evaded both conventional and integrated pest management (IPM) strategies in many countries^21^. Options include the entomopathogenic fungi (EPF) *Metarhizium anisopliae* s.l. and *Beauveria bassiana*. However, EPF have been found to be most effective against eggs (with mortality between 79 and 87%) and newly emerged larvae (50–70% for *B. bassiana*) with a significant drop in efficacy reported when tested against 2nd instar larvae (< 10% for *B. bassiana*)^22^. This is consistent with most pest control approaches, with earlier stage larvae more susceptible to EPF^12^. Although EPF use as biopesticides are promising and has many advantages, commercialisation has suffered from the increased lag time between application and effective population control when compared to conventional synthetic pesticides^23^.

Botanical insecticides offer another potential environmentally benign pest control option for FAW. Recently, Rioba and Stevenson^24^, reported efficacy results for 69 plant extracts against FAW, with high mortality reported from multiple plant species. Chawanda et al.^25^ also report effective control of FAW using soil and plant extracts. While this imposes greater costs and additional labour, they have reduced impacts on beneficial insects compared to synthetic pyrethroids^26^ and are economically viable^27^. More generally, botanical insecticides require higher frequency applications than synthetic pesticides due to the active components being UV labile^28–30^. One of the oldest botanical insecticides is pyrethrum, which continues to be the most prevalent biopesticide on the market^31^, which, like other botanical pesticides, is UV-labile^30^. Although this non-persistent nature of pyrethrum is in some ways a drawback, it has a reduced environmental damage impact profile compared to conventional treatments^32,33^ and additionally pyrethrum has a lower mammalian toxicity than synthetic pyrethroids^34^.

To mitigate the inherent limitations of EPF and botanical insecticides, attempts have been made to combine these treatments^35–39^ against a range of pest species, using a variety of botanicals and EPF strains. However, these have had contrasting results. Generally, combination treatments can be described as either antagonistic, additive, or synergistic with a focus on the performance of the EPF while in the presence of the botanical insecticide.

In the present study, we assessed the efficacy of the combination of pyrethrum and *B. bassiana* against FAW. In vitro radial growth assays were also performed to assess the compatibility of *B. bassiana* with pyrethrum, with the potential for this combination treatment to be utilised in the control of FAW discussed.

## Materials and methods

### Insect rearing

FAW were collected from wild populations around Fujian, China and brought to Natural Resources Institute (University of Greenwich, UK) for rearing and experimental work. Larvae were maintained on potted barley plants (*Hordeum vulgare* L.) and supplemented with an artificial diet in a controlled environment laboratory at 27 °C ± 2 °C, on a 12 h, L:D cycle. The artificial diet consisted of soya flour (100 g), brewers’ yeast (15 g), l-ascorbic acid (1.5 g), methyl 4-hydroxybenzoate (1 g), sorbic acid (0.5 g), formaldehyde (1.2 mL), Agar (6 g), deionised water (375 mL). At instar 6 or 7 the insect pupates for 7–10 days, at which point they were transferred to a BugDorm™ (47.5 × 47.5 × 93.0 cm, 165 µm mesh) with a relative humidity of 60–80%. Adults that emerged were provided with a 20% sucrose solution as a food source. Potted barley plants were placed in the bug dorm for natural oviposition and were replaced at timed intervals with the previous plant transferred to a sealed plastic container (15 × 28 × 9 cm), ensuring approximately equivalent age of hatched larvae. Final determination of FAW instar for all experiments was achieved by measuring head capsule width for each individual larva^40^.

### *B. bassiana* conidia suspensions

Commercially available *B. bassiana* Naturalis-L (strain ATCC 74040) was obtained from Fargro Ltd, UK. This strain was cultured on Potato Dextrose agar (PDA) in 90 × 15 mm Petri dishes and maintained at 25 ± 2 °C in darkness for 7–14 days until sporulation. A suspension of conidia was harvested by pipetting 10 mL of 0.01% Triton X-100 (BDH Chemicals, VWR, Poole, UK), onto the PDA plate containing conidia and was gently agitated using an L-shaped spreader (Fisher Scientific) for approximately 2 min. The suspension was then filtered through two layers of sterile milk filter paper (Goat Nutrition LTD, Kent, UK) to remove mycelial fragments.

The conidial suspension was vortexed for 2 min using a vortex mixer (Whirlimixer, Fisher Scientific^©^ UK Ltd, Leicestershire, UK), then diluted 1 in 10 in 0.01% Triton X-100. Conidia were counted under a light microscope (Dialux 20L EB, Leitz microsystems, Germany) using an improved Neubauer haemocytometer (Hawksley, Sussex, UK). At least three ‘C’ cell frames were counted, with individual counts showing between 20 and 100 conidia per square, this process continued until at least 300 conidia had been counted^41^. The mean number of conidia per square was determined and the concentration of conidia calculated. Aliquots of this stock solution were then diluted to the required concentration in 0.01% Triton X-100 for subsequent experiments.

### Pyrethrum quantification

Pyrethrum was provided by AgroPy (AgroPy Ltd., UK, Batch No: 20190101) as a refined non-commercial solution and compared with an analytical standard purchased from Sigma Aldrich. This refined product was used to reduce any confounding effects of synthetic synergists such as piperonyl butoxide (PBO) and stabilizers such as butylated hydroxy toluene (BHT) which are used in commercial formulations^42^.

The concentration of pyrethrins in the AgroPy product was determined using an Agilent LC–MS system (Agilent Technologies, Santa Clara, United States) consisting of a 1260 series quaternary pump, 1260 series autosampler, 1200 series column oven, 1200 series photodiode detector and an LC/MSD XT single quadrupole mass spectrometer.

The pyrethrum solution was injected as a 10 μL aliquot onto a Waters X-Select T3 column (250 mm × 4.6 mm i.d., 3.5 μm particle size) at 25 °C with a guard column and pyrethrins separated with a flow rate of 1 mL min^−1^, and mobile phases; A (100% H_2_O), B (100% Acetontitrile) and C (1% formic acid in Acetonitrile). The solvent ratio, 27/68/5 (A/B/C), was held for 2 min, raised to 5/90/5 over 22 min (24 min total), followed by wash and re-equilibration steps.

Individual pyrethrins in each sample were identified by comparing their retention times and UV and mass spectra with those of a 52% pyrethrum standard (sum of pyrethrins; Sigma-Aldrich, UK). Quantitative determination of the target compounds in the extracts was performed using external calibration curves at 225 nm on a 5-point calibration of 0.125, 0.25, 0.5, 1 and 2 mg mL^−1^ (total weight of pyrethrins mL^−1^). The experimental product was diluted in 0.01% Triton-X-100 to make a stock solution (5000 ppm). The subsequent concentrations of pyrethrum for treatments were made by dilution of this stock solution with Triton-X-100.

### Formulation of combination treatments

The *B. bassiana* stock solution and the 5000 ppm pyrethrum stock solution was prepared as described in “B. bassiana conidia suspensions” and “Pyrethrum quantification”. For treatments including *B. bassiana*, serial dilutions were made from a 1 × 10^6^ conidia mL^−1^ solution, which was attained via dilution from the stock solution. The process of serial dilutions involved vortex mixing the solution and dispensing 1 mL into a 10 mL sterile vial and diluting with 0.01% Triton-X-100 to achieve a 10 mL final volume. This process was repeated until the desired final concentrations of 1 × 10^5^ and 1 × 10^4^ conidia mL^−1^ of *B. bassiana* were achieved. The pyrethrum was dispensed directly into the sterile vials from the stock solution to achieve the desired concentrations after dilution to 10 mL, 50 µL for 25 ppm and 200 µL for 100 ppm. For the combination treatments, the same process was followed with both treatments combined before dilution to 10 mL with Triton-X-100.

### Mortality assays

The effect of products was determined using a topical bioassay, a method chosen because both Naturalis-L and pyrethrum insecticides are typically applied via traditional spraying methods. The subsequent methodology, which was used for all mortality assays, has also been previously used for accurate topical exposure of pesticides when examining effects of synthetic pesticides and biopesticides^43,44^.

#### General procedure for mortality assays

Ten third instar larvae per treatment were placed in a Petri dish and cooled on ice for 10 min before application. All treatments were applied as 4 mL aliquots onto prepared larvae using a Potter air atomising spray tower (Burkhard Agronomic Instruments, UK) at 5–10 bar pressure (0.34 atm). The tower was cleaned with 70% EtOH solution and 0.01% Triton between treatments.

Three 1 cm^3^ portions of artificial feed were placed in each Petri dish (90 × 15 mm) containing the treated FAW. These dishes were sealed with Parafilm and kept in an incubator at 25 ± 2 °C, relative humidity 60–80%, and a 12L:12D photoperiod for 24 h. Subsequently, the FAW were separated into individual 1 oz portion control pots (Go-Pak UK Ltd, UK). A 1.5 cm^3^ portion of artificial feed was placed in each pot before its lid was applied. The pots were arranged in a matrix and kept in an incubator under the same conditions as previously described for insect culturing.

Larval mortality was checked at different time points for treatments and is detailed in the following sections. Any dead larvae were transferred to damp filter paper and sealed with Parafilm within a Petri dish for assessment of hyphal growth 14 days after death.

#### FAW mortality assays for *B. bassiana* only treatments

Mortality assays were conducted to compare efficacies of different doses of *B. bassiana* against FAW. This data was used to calculate both dose response curves and median lethal dose rates at day 14, as well as to identify suitable doses for later use in combination treatments. Median lethal dose survival assays for *B. bassiana* were performed using concentrations of 1 × 10^3^, 1 × 10^4^, 1 × 10^5^, 1 × 10^6^ and 1 × 10^7^ conidia mL^−1^, with a control treatment of 0.01% Triton*.* All treatments were tested, each with 10 FAW for a given replicate to ensure there was no bias introduced by a specific FAW cohort. The experiment was repeated 3 times, providing a total of 30 individuals tested for each treatment. Mortality was recorded 1, 2, 4, 7, 10 and 14 days after exposure.

The DRC package in R was used to fit dose response curves and estimate the proportional survival of larvae treated with *B. bassiana* at day 14^45^. The dose-responses models were selected by comparing the fit of commonly used dose response models (Log-logistic models, Weibull-1 and Weibull-2) to the data using a goodness-of-fit test, selecting the model with the highest p-value.

#### FAW mortality assays for pyrethrum only treatments

Mortality assays were conducted to compare efficacies of different doses of pyrethrum against FAW. This data was used to calculate both dose response curves and median lethal dose rates at day 14, as well as to identify suitable doses for later use in combination treatments. Median lethal dose survival assays for pyrethrum were conducted with ppm concentrations of 50, 100, 200, 400 and 800 and a 0.01% Triton control. All treatments were tested, each with 10 FAW for a given replicate to ensure there was no bias introduced by a specific FAW cohort. The experiment was repeated 3 times, providing a total of 30 individuals tested for each treatment. Mortality was recorded 1, 2 and 4 days after exposure.

The DRC package in R was used to fit dose response curves and estimate the proportional survival of larvae treated with *B. bassiana* at day 4^45^. The same statistical models were used to analyse survival as described in “FAW mortality assays for B. bassiana only treatments”. FAW mortality assays for combination treatments.

#### FAW mortality assays for combination treatments

Mortality assays were conducted to compare efficacies of different doses of pyrethrum and *B. bassiana*, individually and combined against FAW. This data was used to generate Kaplan–Meier survival curves, calculate differences between expected and observed mortality, and assess the effect of the combination (antagonistic, additive, or synergistic). The combination mortality treatments were applied in concentrations based on the levels of mortality observed in the median lethal dose survival assays. The concentrations chosen for these treatments were those that had resulted in a low (approximately LC_20_) and medium (approximately LC_40_) levels of mortality. These concentrations (ppm = pyrethrum; conidia mL^−1^ = *B. bassiana*) were a 0.01% triton control, 50 ppm, 100 ppm, 1 × 10^–4^ conidia mL^−1^, 1 × 10^–5^ conidia mL^−1^, 50 ppm + 1 × 10^–4^ conidia mL^−1^, 100 ppm + 1 × 10^–4^ conidia mL^−1^, 50 ppm + 1 × 10^–5^ conidia mL^−1^, 100 ppm + 1 × 10^–5^ conidia mL^−1^ (Table 1). All treatments were tested, each with 10 FAW for a given replicate to ensure there was no bias introduced by a specific FAW cohort. The experiment was repeated 4 times, providing a total of 40 individuals tested for each treatment. FAW mortality was recorded 1, 2, 4, 7, 10 and 14 days after exposure to the treatments.

**Table 1:** Expected versus observed corrected mortality at day 14 for combination treatments.

Pyrethrum (ppm)	*B. bassiana* (conidia mL^−1^)	Observed proportional mortality (median ± quartiles)^a^	Observed corrected mortality (median ± quartiles)^b^	Expected corrected mortality (median ± quartiles)^c^	Effect type^d^
Control	Control	0.20 (0.18–0.23)	–	–	
25	–	0.30 (0.28–0.35)	0.12 (0.08–0.19)	–	
100	–	0.40 (0.40–0.50)	0.29 (0.22–0.44)	–	
–	1 × 10^4^	0.50 (0.33 -0.65)	0.42 (0.18–0.56)	–	
–	1 × 10^5^	0.55 (0.48–0.60)	0.44 (0.38–0.46)	–	
25	1 × 10^4^	0.40 (0.35–0.43)	0.20 (0.13–0.28)	0.54 (0.23–0.71)	21.41 (Antagonistic)
100	1 × 10^4^	0.60 (0.48–0.70)	0.53 (0.37–0.63)	0.68 (0.39–0.83)	3.76 (Additive)
25	1 × 10^5^	0.60 (0.50–0.60)	0.50 (0.34–0.51)	0.52 (0.49–0.54)	0.08 (Additive)
100	1 × 10^5^	0.75 (0.60–0.83)	0.71 (0.50–0.78)	0.63 (0.60–0.68)	1.02 (Additive)

^a^n = 4, ^b^calculated following Schneider–Orelli’s formula^48^, ^c^1 − ((1 − A) × (1 − B)), where A and B are the corrected proportional mortality due to EPF and pyrethrum treatments alone. ^d^Calculated from Chi-squared test against expect mortality from individual treatments.

Kaplan–Meier survival curves were generated for each treatment in R^46^. Curves were compared through a global log rank test followed by pairwise tests with Bonferroni adjustment to P values for multiple comparisons^47^. To further examine possible antagonistic or synergistic interactions between treatments in proportion of FAW killed after 14 days, data were first corrected for control mortality in each replicate following Schneider–Orelli’s formula^48^. Expected mortality for each combination treatment were calculated as 1 − ((1 − A)*(1 − B)), where A and B are the corrected proportional mortality due to EPF and pyrethrum treatments alone. A Chi-squared test was performed to identify significant differences between observed and expected mortalities^49^. Chi-squared values were calculated using the observed corrected percentage mortality (M_o_) and expected corrected percentage mortality (M_e_) using the following equation ${x}^{2}=\frac{{({M}_{o}-{M}_{e})}^{2}}{{M}_{e}}$. These values were compared to the Chi-squared critical value for 1° degree of freedom at 0.05 significance (3.841). Values higher than this indicates synergistic or antagonistic interactions, while values lower than this suggests additive effects.

Binomial logistic regression in R was used to compare proportions of dead FAW which produced hyphae between treatments^50^.

### *Beauveria bassiana* radial growth assays

Radial growth assays were performed to quantify any inhibition in *B. bassiana* growth resulting from application of different concentrations of pyrethrum.

Conidia suspensions of Naturalis-L were prepared by diluting the commercial product in 0.01% Triton X-100 to give a final concentration of 1 × 10^7^ conidia mL^−1^. Then 100 µl of the suspension was spread over a PDA plate using a sterile ‘L’ shaped spreader and left for 48 h at 25 °C.

PDA plates were prepared containing different concentrations of pyrethrum (800 ppm, 400 ppm, 200 ppm, 100 ppm, 50 ppm, 0 ppm) by adding the appropriate volumes of the pyrethrum stock (5000 ppm) to cooled molten PDA just prior to pouring.

A metal cork-borer was used to take 1 cm plugs from the prepared Naturalis plate, which were then inverted into the centre of a 90 mm Petri dish containing the prepared PDA. Three plates were prepared for each concentration of pyrethrum at each incubation temperature (15 °C, 20 °C, 25 °C). This range of temperatures was selected as it has been used previously to measure the effect of herbicides and fungicides on mycelial growth of *B. bassiana* (strain ATCC 74040)^51^. Radial growth on these plates was monitored by measuring the pre-drawn x and y axis then calculating the mean growth for each plate. Growth was measured at least once a week for either 33 days or until the growth had reached the edge of the plate. As such, for plates incubated at 25 °C growth was last measured at day 14, for 20 °C at day 19, and for 15 °C at day 33. The whole experiment was repeated three times (nine plates per temperature treatment).

General linear mixed models (GLMMs) were used to examine the effect of pyrethrum concentration on radial growth rate at each temperature tested^52^. Radial growth was entered as the dependent variable, with time (days) entered as a continuous independent variable and pyrethrum concentration entered as a six-level factor. Effect of pyrethrum concentration on growth rate was tested through significance of a time by pyrethrum interaction in the model. Replicate and plate within replicate were entered as random effects. Significance of the time by pyrethrum concentration interaction in each model was assessed through χ^2^ tests of change in residual deviance following deletion from the model. Post hoc comparisons between growth rates at each pyrethrum concentration were made using Tukey’s tests (Lenth et al. 2022)^50^. Modelling was conducted in R 4.2.1^53,54^.

## Results

### FAW median lethal dose survival assays for individual treatments

#### FAW median lethal dose survival assays for *B. bassiana*

**Figure 1:**
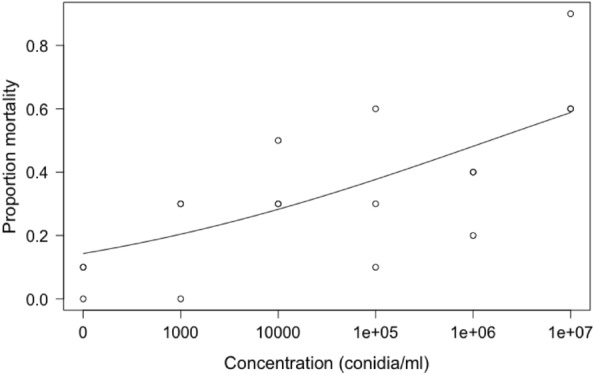
Proportion mortality of FAW (n = 30 for each concentration) following treatment with *B. bassiana* (ATCC 74040) on day 14 after treatment. A two-parameter log-logistic model is fitted to the responses.

There was a positive relationship between FAW mortality and the concentration of *B. bassiana* applied to larvae (Fig. 1). The response was best described by a two-parameter log-logistic function^55^. On day 14 after treatment, the mean mortality of FAW treated with the highest concentration (1 × 10^7^ conidia mL^−1^) of *B. bassiana* was 70% and lowest concentration (1 × 10^3^ conidia mL^−1^) was 20%. The median lethal dose was calculated on day 14 after treatment and found to be 1.48 × 10^6^ conidia mL^−1^ which was higher than the recommended field application concentration of 3.45 × 10^4^ conidia mL^−1^, indicating that *B. bassiana* may not be at peak efficiency as a control agent when used in isolation at field realistic concentrations.

#### FAW median lethal dose survival assays for pyrethrum

**Figure 2:**
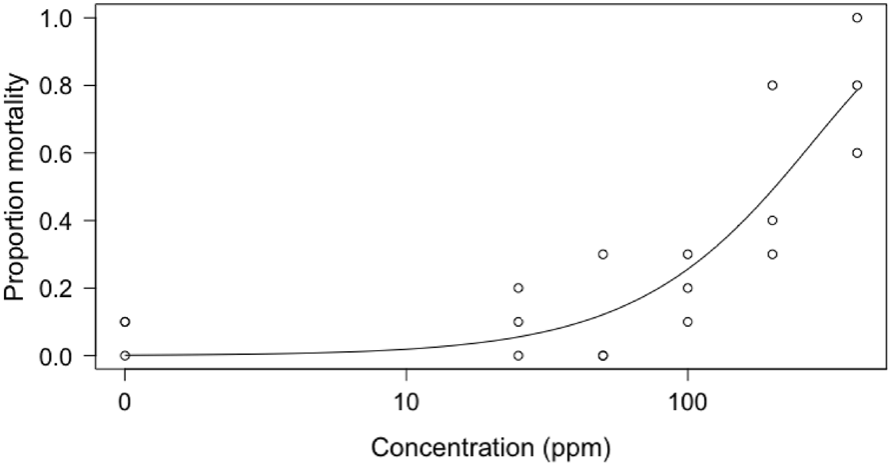
Proportion mortality (n = 30 for each concentration) of FAW following treatment with pyrethrum on day 14 after treatment. A two-parameter Weibull model is fitted to the responses.

There was a positive relationship between FAW mortality and the concentration of pyrethrum applied to larvae (Fig. 2). The response was best described by a two-parameter Weibull function^55^. On day 14 after treatment the mean mortality of larvae treated with the highest concentration of pyrethrum (400 ppm) was 80% and with the lowest concentration (25 ppm) was 10%. The median lethal dose on day 14 was found to be 193 ppm.

### FAW mortality assay combination treatments

**Figure 3:**
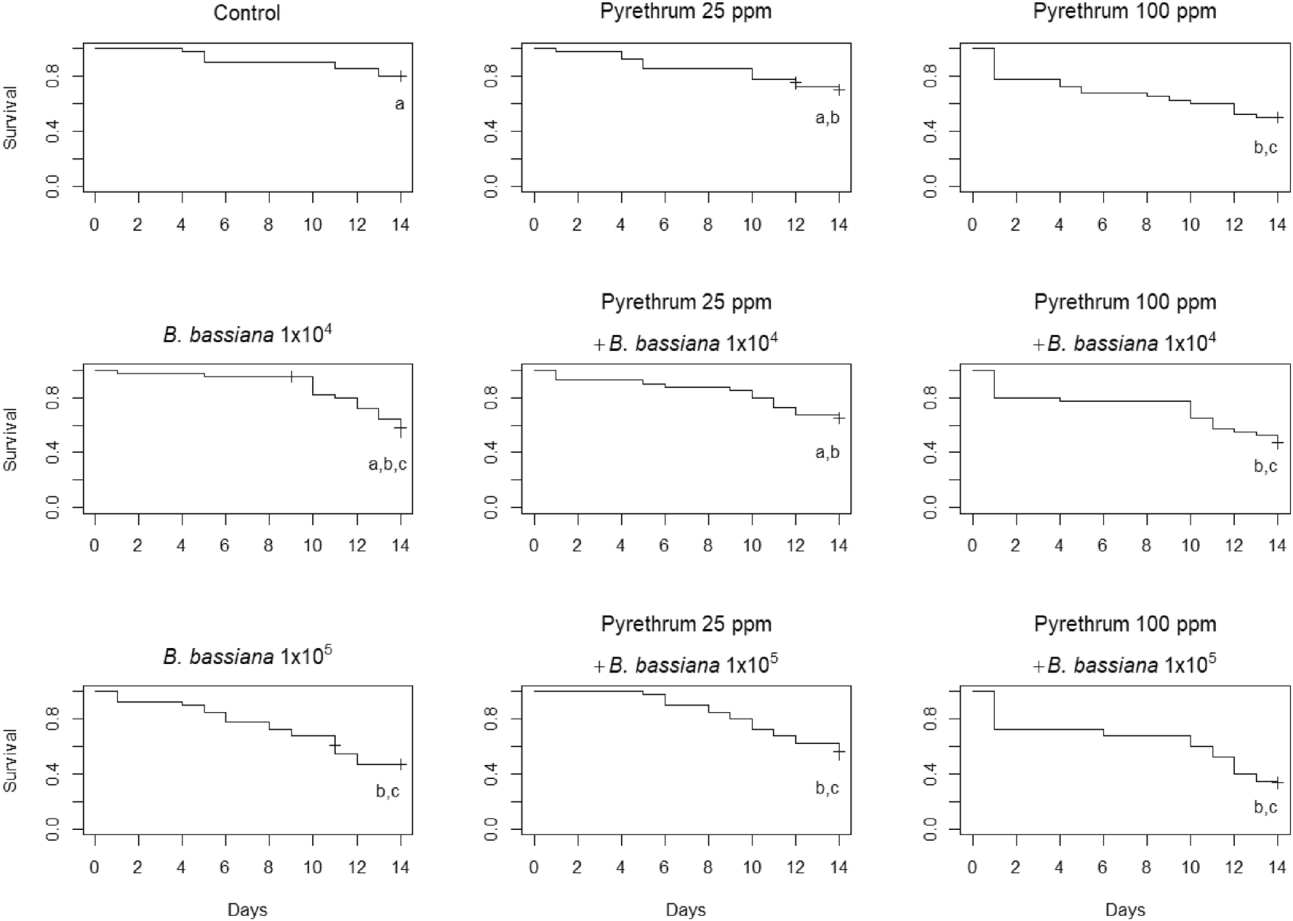
Kaplan–Meier survival curves of effects of pyrethrum (parts per million) and *B. bassiana* (conidia mL^−1^) combination treatments on *S. frugiperda* survival. Different letters indicate significance differences between curves (log-rank test with Bonferroni corrections for multiple comparisons).

An overall significant difference was found between survival curves of FAW exposed to different individual and combination treatments (χ^2^ = 27.2, df = 8, P < 0.001, Fig. 3). Control mortality (Fig. 3, top left), was characterised by a linear decline in survival, with mortality reaching 20% by day 14. Pairwise significant differences (log rank test with Bonferroni correction, P < 0.05) were identified between the control treatment and pyrethrum at 100 ppm (Fig. 3, top right), and the control treatment and *B. bassiana* at 1 × 10^5^ conidia mL^−1^ (Fig. 3, bottom right). The survival curve for the intermediate combination treatment (pyrethrum at 100 ppm plus *B. bassiana* (1 × 10^4^ conidia mL^−1^), Fig. 3, middle) was not significantly different from the control. None of the other combination treatments resulted in significantly different survival compared to pyrethrum 100 ppm and *B. bassiana* (1 × 10^5^ conidia mL^−1^) presented individually (Fig. 3).

Survival curves for treatments containing pyrethrum (100 ppm) were characterized by a 20% drop in mortality at day 1 (Fig. 3, right hand side). However, decline in daily survival then appeared reduced (compared to other treatments) until day 10.

Comparison of expected versus observed corrected mortality for combination treatments suggest there may be some additive and antagonistic interactions between the two biopesticides (Table 1). When pyrethrum was applied at 25 ppm in combination with *B. bassiana* at 1 × 10^4^ conidia mL^−1^, observed corrected mortality was significantly lower than expected for the combination treatments (chi-squared test, P < 0.05), suggesting that there may be antagonistic interactions between these treatments. However, when *B. bassiana* was applied at 1 × 10^4^ conidia mL^−1^ with 25 ppm pyrethrum, 1 × 10^5^ conidia mL^−1^ with 25 ppm pyrethrum or 1 × 10^5^ conidia mL^−1^ with 100 ppm pyrethrum, corrected mortality for the combination treatment was not significantly different from the expected mortality. Therefore, at these concentrations *B. bassiana* and pyrethrum had an additive effect on FAW mortality (Table 1).

### Hyphal growth on insect surface

The percentage of dead FAW producing hyphae in each treatment varied between 40 and 78% (Table 2). None of the dead FAW which had not been treated with EPF (control, 25 ppm pyrethrum and 100 ppm pyrethrum treatments) produced hyphae 14 days after exposed to treatment or 7 days after death (Table 2). No significant overall difference was found between the remaining EPF and EPF plus pyrethrum treatments in proportion of dead FAW producing hyphae (binomial logistic regression, χ^2^ = 8.0, df = 5, P = 0.16).

**Table 2:** Percentage of dead FAW treated with *B. bassiana* and pyrethrum which produced fungal hyphae.

*B. bassiana* (conidia mL^−1^)	Pyrethrum (PPM)	Total dead FAW	No. dead FAW which produced hyphae	Percentage dead FAW which produced hyphae (%)
0	0	8	0	0
0	25	13	0	0
0	100	20	0	0
1 × 10^4^	0	19	10	53
1 × 10^4^	25	15	6	40
1 × 10^4^	100	23	16	70
1 × 10^5^	0	21	12	57
1 × 10^5^	25	20	14	70
1 × 10^5^	100	27	21	78

### *B. bassiana* radial growth assay

**Figure 4:**
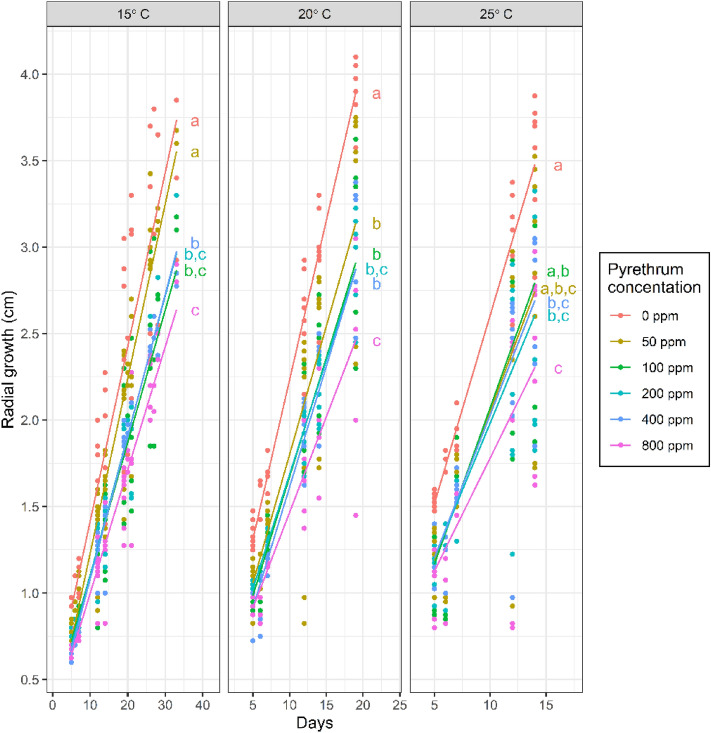
Radial growth rates of EPF treated with pyrethrum at five temperatures. Lines are predictions of fixed effects from generalized linear models. Treatments labelled with different letters have significantly different radial growth rates (Tukey’s tests on line slopes, P < 0.05).

Significant effects of pyrethrum on EPF growth rate were found at all temperatures tested (GLMMs: df = 5, P < 0.001, 15 °C: χ^2^ = 102.7, df = 5, P < 0.001; 20 °C: χ^2^ = 75.2, df = 5, P < 0.001, 25 °C: χ^2^ = 27.5, df = 5, P < 0.001, Fig. 4). EPF growth rate was significantly (Tukey’s test, P < 0.05) reduced compared to the control (0 ppm pyrethrum) at all pyrethrum concentrations except: 50 ppm (15 °C), 50 ppm (25 °C) and 100 ppm (25 °C) (Fig. 4).

## Discussion

Used in isolation pyrethrum showed good efficacy against FAW with a median dose of 193 ppm which is approximately half the recommended concentration for field application (364 ppm), indicating that pyrethrum is likely an effective biopesticide against 1st to 3rd instars. Conversely, the strain of *B. bassiana* used here did not give high levels of mortality with median lethal dose, when tested in isolation, of 1.48 × 10^6^ conidia mL^−1^, which was orders of magnitude higher than the recommended field application of 3.45 × 10^4^ conidia mL^−1^, with a high degree of variation between replicates seen. These findings do indicate that *B. bassiana* in isolation could be an ineffective population control technique for FAW larvae. These findings were in keeping with the results reported by *Akutse et al.^22^ who found that several strains of *B. bassiana* were ineffective on FAW larvae past the 2nd instar with similar findings for *M. anisopliae*. This low efficacy from EPF strains is likely linked to the relatively short duration of instars, an inherent defence mechanism of FAW larvae. Each time the larvae progress to a new instar it offers a chance for any attached infective conidia to be “moulted off” thus rendering the fungi ineffective^56^.

When used in combination, the biopesticides appear to have an antagonistic relationship at the lowest concentrations of *B. bassiana* and pyrethrum (1 × 10^4^ conidia mL^−1^ with 25 ppm) and an additive effect for the other 3 combined treatments (1 × 10^4^ conidia mL^−1^ with 100 ppm and 1 × 10^5^ conidia mL^−1^ with 25 ppm and 100 ppm pyrethrum, Table 1). However, some caution should be applied to these results due to the low number of replicates in this study, especially for the combination treatment of 1 × 10^4^ conidia mL^−1^ with 100 ppm, which had lower observed mortality than expected although this was not significant. However, it does seem clear that there are some negative interactions affecting efficacy when combining the two biopesticides, *B. bassiana* and pyrethrum, for control of FAW.

The reason for an antagonistic effect could be attributed to impediment of growth which was seen in the radial growth assays where the growth rate of *B. bassiana* was significantly reduced by pyrethrum. However, the concentrations of pyrethrum used, which went as high as double the recommended concentration for field use (800 ppm), were not enough to completely impede the fungus in these trials. If a minimum dose of colony forming units (CFU) is required for effective *B. bassiana* control, and the pyrethrum only causes a proportional reduction in colony growth, then it may be that the EPF needs to exceed a critical threshold before the total CFU are adequate for the benefits to be seen. The Kaplan–Meier survival curves also appear to show this effect, with the pathogenic effect of the EPF seemingly being ‘delayed’, which was especially evident when comparing the highest concentration of *B. bassiana* tested in isolation (1 × 10^5^ conidia mL^−1^) with the equivalent concentration combined with the higher pyrethrum (100 ppm). This combined treatment had a mortality effect at day 1 (likely due to the pyrethrum) but with little additional mortality seen until day 10, which is in contrast to the *B. bassiana* alone which showed a more continuous effect from day 4, but did not have the same initial mortality effect. Litwin et al.^57^ identified that pyrethroids changed the phospholipid profile of *B. bassiana* and had a detrimental effect by increasing the cell membrane permeability, accumulation of the pyrethroids within cells, and an overall increase in oxidative stress. Furthermore, Litwin et al. found a decrease in production of oosporein (a secondary metabolite toxin produced by *B. bassiana*), which is known to have immunomodulation properties^58^. Although not perfectly analogous, pyrethrum and pyrethroids have similar modes of action and thus it is reasonable to assume that pyrethrum would have a similar effect on *B. bassiana* as the pyrethroids tested by Litwin et al. (λ-cyhalothrin, α-cypermethrin, and deltamethrin). A reduction in oosporein production would at least partially explain why there is an apparent delay to the pathogenic effect of *B. bassiana*, as it would take longer for the fungi to overcome the immune system of the insect. However, this effect did not seem to impede further propagation of the fungi, with no significant differences seen in the proportion of cadavers producing hyphae between the *B. bassiana* alone and the equivalent *B. bassiana* + pyrethrum treatments. This secondary cycling of the pathogen within the pest population is a key benefit of EPF’s use as a biopesticide and any reduction in this effect would result in reduced efficacy.

The beneficial effect of the combined treatment is likely linked to the short period of time between instars mentioned previously^53^. Stunting of the development of FAW has previously been shown in FAW larvae when exposed to sublethal concentrations of synthetic pyrethroids^59^. When used in combination with EPF, any stunting effect would allow more time for the EPF infection to progress beyond the point where the moulting remains effective in shedding fungi. Additionally, this effect will increase the spore to larval size ratio compared to larvae that are not stunted thus increasing the former’s susceptibility. Taken in conjunction, these factors could go some way to explaining some of the conflicting results seen in both our own work, and results seen in previous work on similar interactions between pathogens^60^. We demonstrate that combining EPF and a botanical pesticide has potential in control solutions by providing a greater level of mortality than either component independently. However, the work supports previous findings that the compatibility between the components varies between EPF species^61^ and isolates^62^, botanical component used^37^, and, as our own work highlights, the dosage applied.

Irrespective of the classification of the interactions between the biopesticides used here (synergistic, additive or antagonistic), this type of combination has potential benefits for control of FAW. Similar levels of mortality were found at Day 14 in one of the combined treatments (100 ppm pyrethrum and 1 × 10^5^ conidia mL^−1^) to the 400 ppm pyrethrum in isolation which is roughly equivalent to the recommended field application of 364 ppm. This result represents a significant decrease in the application levels of the insecticidal component, which in this study was pyrethrum, which is already environmentally preferable to synthetic pyrethroids due to the higher decay rate in field conditions and lower mammalian toxicity^30,34,63^. Furthermore, the natural source of pyrethrum, *Tanacetum cinerariifolium*, can be grown locally in many of the environments where FAW is considered an invasive pest species^64^. Reducing quantities of pyrethrum required for effective control may not only reduce the impact on non-target species but may also lead to more economic solutions for growers.

The additive effect seen here, at one concentration of *B. bassiana*, does indicate that combining EPF with pyrethrum may have merit. Further work could be undertaken to identify the optimal dose rate for the two treatments and investigate the merit of applying *B. bassiana* at different time periods after applying pyrethrum. Alternately, a more effective EPF strain against FAW, which has growth less impeded by pyrethrum, could allow for synergy and, therefore, a more effective IPM approach.

## Conclusion

Dose-dependent interactions between *B. bassiana* and pyrethrum were observed in this study. A delay in the pathogenicity of *B. bassiana* against FAW is not universally beneficial or detrimental to pest control strategies. However, the results shown here highlight several of the beneficial effects that this approach can have, the immediate knockdown from the botanical pesticide combined with the longer lasting effect from the EPF and a reduced environmental impact from the lower concentration of insecticidal component required.

## Data Availability

The datasets used and/or analysed during the current study available from the corresponding author on reasonable request.
